# Prophylactic role of artemisinin in modulating FGFR3, HRAS, and TP53 to prevent early-stage urothelial carcinoma in BBN-induced mouse models

**DOI:** 10.1186/s12896-025-01039-4

**Published:** 2025-09-17

**Authors:** Silvia Botrous, Ayaat Elmaghraby, Samar El Achy, Yehia Mustafa, Salah Abdel-Rahman

**Affiliations:** 1 Department of Genetics, Faculty of Agriculture, Alexandria University, Alexandria, Egypt; 2 Department of Nucleic Acids Research, Genetic Engineering and Biotechnology Research Institute (GEBRI), City of Scientific Research and Technological Applications (SRTA-City), Alexandria, Egypt; 3 Department of Surgical Pathology, Faculty of Medicine, Alexandria University, Alexandria, Egypt

**Keywords:** Artemisinin, Prophylactic agent, Urinary bladder cancer, Urothelial carcinoma, N-butyl-N-(4-hydroxybutyl) nitrosamine (BBN), Protein–protein interaction network, Molecular docking, Gene expression profiling, FGFR3, HRAS, TP53, Early-stage urothelial carcinoma (pT1), Bladder carcinogenesis

## Abstract

**Purpose:**

Urinary bladder cancer remains a significant global health challenge, with effective early preventive strategies urgently needed to reduce incidence and progression. This study explores the prophylactic potential of artemisinin against N-butyl-N-(4-hydroxybutyl) nitrosamine (BBN)-induced early-stage urothelial carcinoma in a mouse model.

**Methods:**

A multidisciplinary approach was used to evaluate artemisinin’s molecular and physiological effects. Techniques included protein–protein interaction (PPI) network analysis, molecular docking, gene expression profiling, histopathological evaluation, and systemic biomarker assessment.

**Results:**

PPI analysis revealed FGFR3, HRAS, and TP53 as central oncogenic drivers. Molecular docking confirmed strong binding affinities of artemisinin to these targets. Prophylactic artemisinin administration significantly downregulated FGFR3 and HRAS while upregulating TP53, indicating early correction of carcinogenic signaling. These molecular changes were associated with preserved bladder and renal histoarchitecture, normalized kidney function markers, and restored hematological profiles, reflecting systemic protection against BBN-induced toxicity.

**Conclusions:**

Artemisinin effectively intercepts bladder carcinogenesis at multiple levels, modulating key genetic pathways and mitigating systemic damage. These findings provide compelling preclinical evidence supporting artemisinin as a promising prophylactic agent for bladder cancer prevention in high-risk populations.

## Introduction

Urinary bladder cancer ranks as the 10th most commonly diagnosed cancer worldwide and the 13th leading cause of cancer-related mortality. By 2040, the global incidence and mortality are projected to reach nearly 981,000 new cases and 392,000 deaths, respectively [1]. Despite this high burden, bladder cancer remains largely preventable due to the presence of modifiable risk factors. Tobacco smoking is the predominant risk factor, accounting for approximately 50% of cases and 37% of bladder cancer-related deaths. Smokers are two to four times more likely to develop bladder cancer than non-smokers [2]. Cigarette smoke contains several bladder-specific carcinogens, such as β-naphthylamine, polycyclic aromatic hydrocarbons, and nitrosamines including N-butyl-N-(4-hydroxybutyl) nitrosamine (BBN). These carcinogens induce inflammation, form DNA adducts, and promote permanent genetic alterations that activate oncogenes or inactivate tumor suppressor genes, thereby driving urothelial carcinogenesis [3].

BBN, a metabolite of N-nitroso-di-N-butylamine present in tobacco smoke, is a potent bladder carcinogen in rodents. Following ingestion, it is metabolized into N-butyl-N-(3-carboxypropyl) nitrosamine (BCPN), which reaches the bladder epithelium and covalently binds to urothelial macromolecules, inducing DNA damage and initiating tumorigenesis [4]. BBN carcinogenicity is mediated through DNA alkylation, oxidative stress, and accumulation of mutations, culminating in muscle-invasive bladder cancer (MIBC) in mice [5]. These BBN-induced tumors closely recapitulate the mutational landscape of human MIBC, particularly alterations in TP53, RAS, and H19 (involved in regulating cell proliferation and tumor progression), with a high mutational burden and basal subtype features [6, 7].

**Fig. 1:**
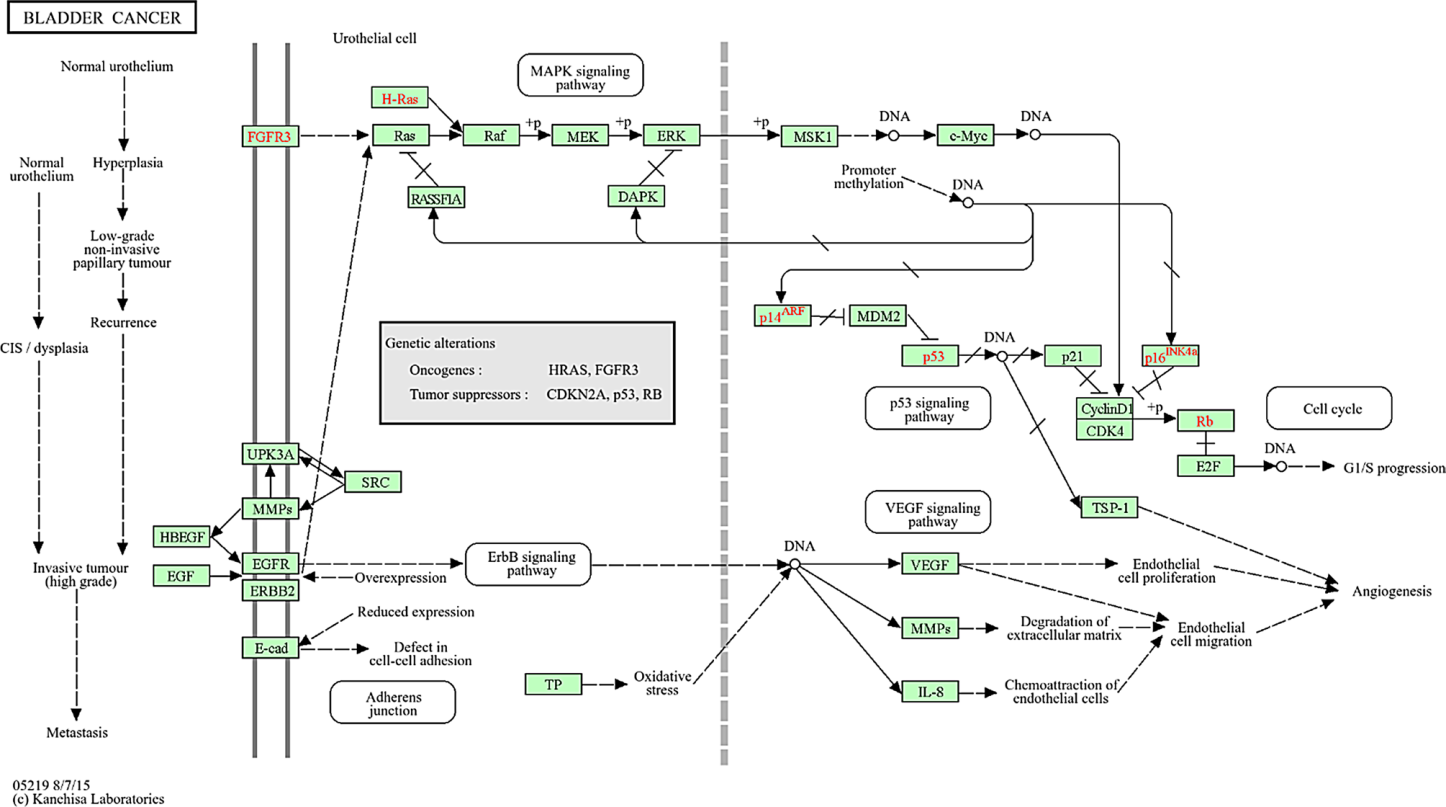
Molecular Pathways in bladder cancer progression. This image illustrates the molecular pathways involved in bladder cancer progression highlighting the transition from normal urothelium to invasive tumors. It emphasizes key genetic alterations in oncogenes (HRAS, FGFR3) and tumor, suppressor genes (TP53, CDKN2A, RB), as well as critical signaling pathways (MAPK, p53, VEGF, ErbB) that regulate essential processes

Key driver mutations in FGFR3, HRAS, and TP53 underlie distinct molecular pathways in bladder cancer. FGFR3 mutations are frequently detected in non-invasive, low-grade papillary tumors (~ 50% of cases) and are associated with favorable clinical outcomes [8–10]. In contrast, HRAS mutations, reported in 0–84% of cases depending on the study population, stimulate urothelial hyperplasia, with a higher prevalence among younger patients, suggesting a role in early disease onset [11–13]. TP53 mutations, found in approximately 50–60% of muscle-invasive or high-risk bladder cancers, impair DNA repair and cell cycle regulation, correlating with aggressive phenotypes and poor prognosis [12, 14]. Collectively, these mutations converge on signaling cascades such as MAPK (involved in cell proliferation, differentiation, and survival signaling), VEGF (promotes angiogenesis and vascular development), and ErbB (involved in cell growth and differentiation), which orchestrate proliferation, angiogenesis, adhesion, and extracellular matrix degradation. Understanding these pathways provides critical insights into bladder cancer development and potential therapeutic targets, as illustrated in Fig. 1 and [15–17].

Among natural compounds investigated for anticancer potential, artemisinin, a sesquiterpene lactone isolated from Artemisia annua, has shown remarkable pharmacological versatility. Originally discovered for its potent antimalarial activity by Nobel laureate Tu Youyou, artemisinin and its derivatives (dihydroartemisinin, artesunate) have since been recognized for diverse biological effects, including anti-inflammatory, antidiabetic, and immunomodulatory properties [18, 19]. More importantly, artemisinin exhibits broad anticancer activities through multi-targeted mechanisms, such as cell cycle arrest, inhibition of angiogenesis and metastasis, induction of ferroptosis, and selective cytotoxicity toward cancer cells [20]. Its anticancer potential has been validated across a wide spectrum of malignancies, including breast, urinary bladder, prostate, ovarian, and lung cancers, as well as in large-scale screening across 55 different cancer cell lines [21–28]. Despite these advances, the prophylactic role of artemisinin remains largely unexplored, particularly in bladder cancer. Given its multi-targeted mechanisms including anti-inflammatory, antioxidant, and iron-dependent cell death pathways artemisinin may interfere with early oncogenic events induced by carcinogens such as BBN, which initiate urothelial mutations in FGFR3, HRAS, and TP53. Therefore, this study represents one of the first attempts to investigate artemisinin as a preventive agent against BBN-induced bladder carcinogenesis, providing a strong rationale for its selection based on both established anticancer activity and potential preventive effects.

Accordingly, the present study aims to assess the efficacy of artemisinin as a prophylactic agent against early-stage urothelial carcinoma induced by BBN in male albino mice and to elucidate its underlying molecular mechanisms through modulation of critical oncogenes (FGFR3, HRAS) and the tumor suppressor gene TP53.

## Materials and methods

### Protein-protein interaction network analysis

To investigate the functional associations and interactions between key genes involved in bladder cancer progression FGFR3, HRAS, TP53, FRS3 (involved in FGF signaling and cell proliferation), RPS6KA2 (regulating MAPK pathway and cell growth), SHC2 (mediates signal transduction from receptor tyrosine kinases), RGL1(regulates small GTPase signaling and cell proliferation), PPP1R13B (modulates apoptosis and interacts with p53 pathway), a protein-protein interaction (PPI) network analysis was performed using the STRING database (version 12.0, https://string-db.org/). The selected genes were input into the STRING platform, and the network was constructed based on various evidence sources, including experimental data and co-expression. The resulting network visually represents the direct and indirect protein associations, providing insights into their potential cooperative roles in bladder cancer pathogenesis.

### Protein sequence alignment

To ensure the suitability of mouse protein structures for molecular docking studies targeting human homologs, the nucleotide and protein sequences of the target genes (HRAS, TP53, and FGFR3) from Mus musculus and Homo sapiens were retrieved in FASTA format from the NCBI RefSeq database (https://www.ncbi.nlm.nih.gov) Pairwise sequence alignments between mouse and human proteins were conducted using the EMBOSS Needle tool (version 6.6.0), available at the European Bioinformatics Institute (EBI) web server (https://www.ebi.ac.uk/jdispatcher/psa/emboss_needle). The alignments utilized the BLOSUM62 substitution matrix with default gap penalties (gap open: 10.0, gap extend: 0.5) optimized to balance sensitivity and accuracy in identifying conserved regions.

### Molecular docking analysis

To investigate the potential prophylactic effects of artemisinin against bladder cancer, molecular docking studies targeted three key regulatory proteins: FGFR3, HRAS, and TP53 from *Mus musculus*. Because high-resolution three-dimensional structures for the mouse proteins were unavailable or incomplete, homologous human protein structures were utilized as surrogates for all three targets to ensure structural accuracy in docking simulations. The canonical isoforms of the mouse proteins and their human homologs were identified, and all relevant gene IDs, accession numbers, and PDB identifiers are provided in Table 1.

**Table 1:** Key molecular targets and homolog structures for FGFR3, HRAS, and TP53 in molecular docking analysis

Gene name	Gene ID	Organism	Gene accession (mRNA)	Protein accession (RefSeq)	Protein accession links	PDB ID (human homolog)	PDB organism	NCBI gene link	Protein link (NCBI)	PDB link
**FGFR3**	14,184	Mus musculus	NM_008010.2	NP_032036.2	GenPept: https://www.ncbi.nlm.nih.gov/protein/Q61851.1 UniProtKB: https://www.uniprot.org/uniprotkb/Q61851/entry	4K33	Homo-sapiens	https://www.ncbi.nlm.nih.gov/gene/14184	https://www.ncbi.nlm.nih.gov/protein/NP_032036.2	https://www.rcsb.org/structure/4K33
**HRAS**	15,461	Mus musculus	NM_008284.3	NP_032310.1	GenPept: https://www.ncbi.nlm.nih.gov/protein/Q61411.2 UniProtKB: https://www.uniprot.org/uniprotkb/Q61411/entry	5P21	Homo-sapiens	https://www.ncbi.nlm.nih.gov/gene/15461	https://www.ncbi.nlm.nih.gov/protein/NP_032310.1	https://www.rcsb.org/structure/5P21
**TP53**	22,059	Mus musculus	NM_011640.3	NP_035770.2	GenPept: https://www.ncbi.nlm.nih.gov/protein/P02340.4 UniProtKB: https://www.uniprot.org/uniprotkb/P02340/entry	1TSR	Homo-sapiens	https://www.ncbi.nlm.nih.gov/gene/22059	https://www.ncbi.nlm.nih.gov/protein/NP_035770.2	https://www.rcsb.org/structure/1TSR

The three-dimensional structures of the human homologs were retrieved from the Protein Data Bank (PDB), while mouse gene and protein sequences were obtained from the NCBI RefSeq database. Protein structures were prepared for docking by removing water molecules and small molecule ligands using the CB-Dock platform (version 2.0; http://cadd.labshare.cn/cb-dock2/), which also generated receptor files suitable for docking. The two-dimensional structure of artemisinin (PubChem CID: 68827) was downloaded from the PubChem database (https://pubchem.ncbi.nlm.nih.gov/) and converted into a three-dimensional conformation optimized for docking using CB-Dock. Active docking pockets were automatically identified by CB-Dock, which recorded spatial locations and cavity sizes for each target protein. Molecular docking simulations between artemisinin and the prepared protein receptors were performed using the AutoDock Vina docking engine (version 1.1.2) embedded in CB-Dock. Binding energies were calculated using the Vina scoring function, and the docking results—including predicted binding poses and interaction sites—were visualized using PyMOL software (version 2.3; https://pymol.org/2/).

### Experimental design

Forty male albino mice (*Mus musculus*), weighing 25–30 g, were randomly selected from the animal house at the Faculty of Pharmacy, Pharos University, Alexandria, Egypt, and subsequently transferred to the Medical Research Institute, Alexandria University. To ensure adaptation, they were initially housed in plastic cages (five per cage) under controlled environmental conditions (temperature: 25 °C, humidity: 50%, and a 12-hour light–dark cycle), with ad libitum access to food and water. Additionally, the mice were monitored daily for any signs of abnormal behavior and were given a two-week acclimatization period.

**Fig. 2:**
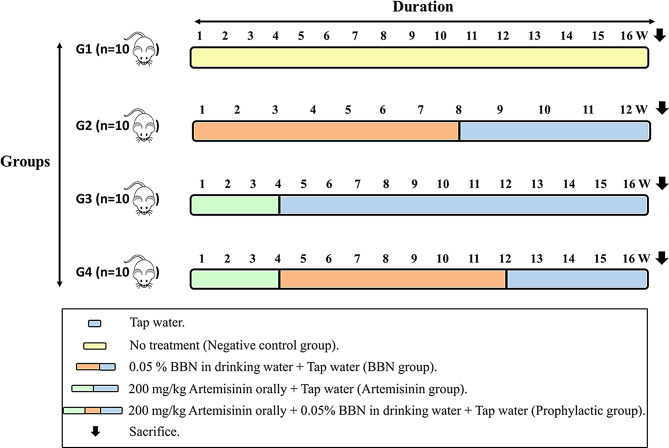
Schematic representation of the experimental design; W: week; n = number of mice

Following acclimatization, the mice were randomly assigned to four groups (*n* = 10 per group), each following a distinct experimental protocol, as illustrated in Fig. 2; Table 2: A negative control group (A group), receiving only tap water throughout the experiment. The BBN group (B group) received tap water containing 0.05% BBN (Sigma–Aldrich, Dorset, UK) for 8 weeks to induce urinary bladder cancer in male albino mice. The BBN water was then replaced with regular tap water for 4 additional weeks. Mice were sacrificed at 0, 4, 8, and 12 weeks to assess tumor development, in accordance with reference [7]. and our previous study [26]., as outlined in Fig. 2. Artemisinin-treated health group (C group) received oral artemisinin at a dose of 200 mg/kg/day for 28 consecutive days to assess its preventive potential, as described in [29] All animals were monitored daily for signs of toxicity, including changes in body weight, food and water intake, activity, grooming, and mortality. Artemisinin at 200 mg/kg was well tolerated, and no adverse effects were observed throughout the study. Prophylactic group (Artemisinin + BBN) received daily artemisinin for 4 weeks prior to BBN exposure, aiming to assess its preventive effect against early-stage urothelial carcinoma. After the 4-week artemisinin regimen, followed by 8 weeks of BBN exposure. This was then succeeded by 4 additional weeks of tap water before sacrifice, as also illustrated in Fig. 2. Prior to euthanasia, the mice were anesthetized via intraperitoneal injection of 150–200 mg/kg amobarbital sodium and euthanized by cervical dislocation. Afterward, kidney and urinary bladders tissues were harvested and sagittally divided into two sections: one was snap-frozen in liquid nitrogen for RNA isolation, while the other was fixed in 10% formaldehyde for paraffin embedding and histological analysis. Notably, all animals successfully completed the 16-week study protocol.

Finally, the study was conducted in strict compliance with ethical guidelines for the care and use of laboratory animals, as approved by the Institutional Animal Care and Use Committee (AU-08-22-07-24-2-96) at the Faculty of Medicine, Alexandria University, Egypt.

### Real-time quantitative PCR (qRT-PCR)

Total RNA was isolated from the urinary bladder tissues of male albino mice using the Gene JET RNA Purification Kit (Geneaid Biotech, Korea). The RNA concentration and purity were assessed using a Nanodrop Spectrophotometer. RNA samples were normalized to ensure uniform concentration across all samples, minimizing the risk of false elevation in gene expression levels. Gene expression of oncogenes (FGFR3 and HRAS), the tumor suppressor gene (TP53), and the reference gene (GAPDH) was quantified using the SYBR Green One-Step qRT-PCR Kit (Enzymomics, Korea) and a Real-Time PCR System (Bio-Rad, USA). Specific primer sequences (forward/reverse) and the corresponding annealing temperatures for each gene are listed in Table 3.

**Table 2:** Experimental animal design

Group	Animals no.	Treatment
A	10	**Negative control group**: (food + water)
B	10	**BBN group**: BBN (0.05%) in drinking water
C	10	**Artemisinin group**: Artemisinin orally (200 mg)
D	10	**Prophylactic group**: Artemisinin (200 mg) + BBN (0.05%)

**Table 3:** Primer sequences for qRT-PCR

Gene	Primer sequence Forward (F) and Reverse (*R*)	Annealing temperature	Reference
FGFR3	F: 5′-ACAGGTGGTCATGGCAGAAGCT-3′ R: 5′-CTCCATCTCAGATACCAGGTCC-3′	60 °C	[30]
HRAS	F: 5′-TCGCACTGTTGAGTCTCGGCAG-3′ R: 5′-TATGCTGCCGAATCTCACGGAC-3′	60 °C	[31]
TP53	F: 5′-TGAAACGCCGACCTATCCTTA-3′ R: 5′-GGCACAAACACGAACCTCAAA-3′	60 °C	[32]
**GADPH**	F: 5′-CCTCGTCCCGTAGACAAAATG-3′ R: 5′-TGAAGGGGTCGTTGATGGC-3′	55 °C	[32]

The qRT-PCR reaction was conducted in a 10 µL mixture, containing 0.5 µL of TOPreal™ One-Step RT qPCR enzyme mix, 5 µL of TOPreal™ One-Step RT qPCR reaction mix (2X), 0.5 µL each of forward and reverse primers (10 pmol), 0–3.5 µL of PCR-grade water, and 50 ng of RNA template. The qRT-PCR program involved one cycle of cDNA synthesis at 50 °C for 30 min, one cycle of enzyme activation at 95 °C for 10 min, followed by 45 cycles of denaturation at 95 °C for 5 s, annealing at 55–60 °C for 30 s, and extension at 72 °C for 30 s.

The specificity of the PCR amplification was verified by analyzing the melting curves, which ensured the amplification of a single PCR product of the correct size. The threshold cycle (Ct) values were determined for each replicate, and the final gene expression values were obtained as the average of three replicates per sample. Gene expression was normalized to GAPDH expression, and the relative expression levels of target genes were calculated using the 2−∆∆Ct method [33]. The formula for analysis was as follows:

ΔCt (calibrator) = Ct (GOI) c – Ct (norm) c.

ΔCt (sample) = Ct (GOI) s – Ct (norm) s.

ΔΔCt = ΔCt (sample) – ΔCt (calibrator).

Fold difference = 2^−ΔΔCt.

Where “calibrator” refers to the negative control group, “norm” represents the housekeeping gene, “GOI” refers to the gene of interest, “S” denotes the sample, and “C” denotes the control. Statistical significance was determined using one-way ANOVA at a significance level of *P* < 0.05.

### Histological analysis

The kidney and urinary bladder tissues from all animal groups (Negative control group, BBN group, Artemisinin group, and Prophylactic group) were harvested and immediately fixed in 10% neutral buffered formalin for 24 h. The tissues were subsequently dehydrated through ascending grades of alcohol (70%, 80%, 95%, and absolute alcohol), cleared in xylene, and embedded in paraffin wax at 60 °C for 1 h. The specimens were sectioned into 5 μm thick slices, mounted onto clean glass slides, and stained with standard H&E. Morphological alterations in the urothelium were examined under a light microscope both before and after development of cancer.

## Biochemical and hematological analysis

### Biochemical assays

Blood samples were collected to assess kidney function by measuring the levels of urea and creatinine. Serum samples were then separated and collected in tubes designed to prevent contamination. These serum samples were analyzed using the Cobas-C and Cobas-E analyzers (Roche, USA).

### Hematological assays

A Complete Blood Count (CBC) was performed using blood samples collected in EDTA-coated tubes, which prevent clotting. The analysis measured key blood components, including hemoglobin (HB), platelet count (PLTs), red blood cell count (RBCs), and white blood cell count (WBCs). These parameters were analyzed using the Swelab Alpha Basic analyzer (Sweden).

### Statistical analysis

Statistical analysis was conducted using SPSS software (Version 22). Group comparisons were performed using one-way ANOVA, followed by multiple comparisons with repeated measures and post-hoc analysis using LSD test to determine statistical differences between groups. Results are presented as means ± standard deviation (SD) from independent biological replicates (*n* = 10 per group). Sample size (*n* = 10 per group) was determined based on previous similar studies to ensure sufficient statistical power. P value of < 0.05 was considered statistically significant. Graphical representations were generated using GraphPad Prism 10.

## Results

### String protein-protein interaction network

**Fig. 3:**
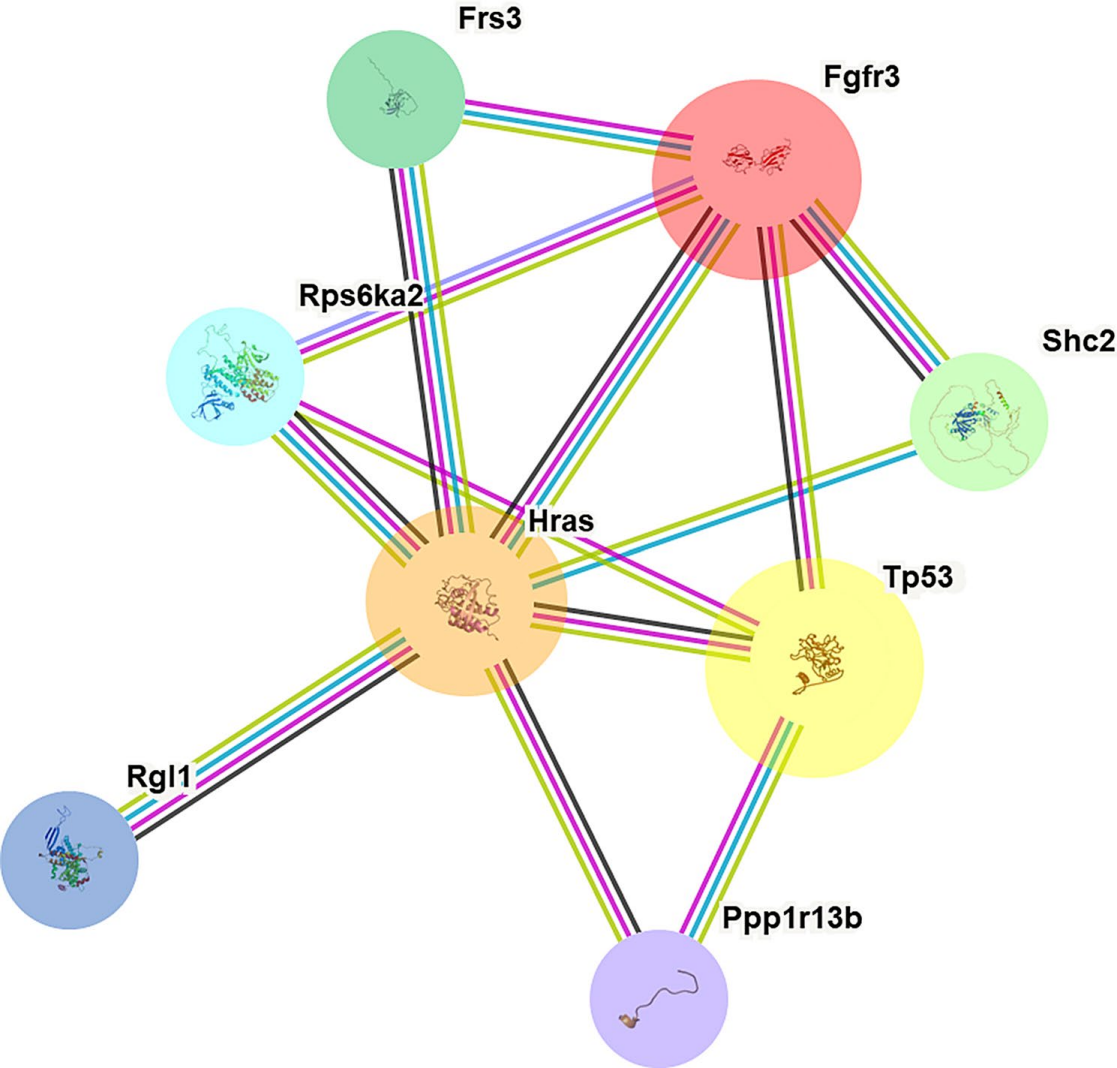
Protein–protein interaction (PPI) network generated using STRING for FGFR3, HRAS, TP53, and their top predicted partners (FRS3, RPS6KA2, SHC2, RGL1, PPP1R13B) in Mus musculus. Each node represents a protein; filled nodes indicate proteins with known or predicted 3D structures, while edges represent protein–protein associations. Edge colors correspond to the type of supporting evidence: **green** = neighborhood, **blue** = co-occurrence, **black** = co-expression, and **pink** = experimental evidence,. HRAS, FGFR3, and TP53 appear as central, highly connected nodes, highlighting their key regulatory roles in oncogenic signaling and apoptosis

The expanded STRING protein-protein interaction (PPI) network analysis elucidated comprehensive functional associations among FGFR3, HRAS, TP53, FRS3, RPS6KA2, SHC2, RGL1, and PPP1R13B (Fig. 3). This network revealed extensive interconnections, highlighting a highly integrated and cooperative role for these genes in bladder cancer pathogenesis. HRAS and FGFR3 emerged as central nodes, demonstrating interactions with multiple network proteins, consistent with their established oncogenic functions. TP53, a crucial tumor suppressor, also exhibited significant connections, underscoring its broad regulatory impact. The inclusion of FRS3, RPS6KA2, SHC2, RGL1, and PPP1R13B further clarified the intricate signaling cascades and regulatory pathways involved. The depicted interactions, indicated by colored lines, reflect diverse evidence supporting these associations, emphasizing the multifaceted molecular mechanisms governing bladder cancer. This interconnectedness further provides a crucial framework for investigating the prophylactic effects of artemisinin against bladder cancer, by elucidating its potential to modulate these complex gene interaction networks.

### Protein sequence alignment

Pairwise sequence alignment of the three target genes (HRAS, TP53, and FGFR3) between mouse and human demonstrated high identity and similarity, confirming their suitability for molecular docking studies. As detailed in Table 4, HRAS showed 99.5% identity and similarity with no gaps and an alignment score of 978.0. TP53 exhibited 76.8% identity and 82.3% similarity with minor gaps (2.3%) and a score of 1558.0. FGFR3 displayed 92.7% identity and 94.9% similarity with minimal gaps (0.7%) and the highest score of 3946.0.

**Table 4:** Pairwise alignment results between gene and protein sequences

Gene	Protein length (aa)	Identity%	Similarity%	Gaps%	Alignment score	Alignment link
**FGFR3**	806	747 / 806 (92.7%)	765 / 806 (94.9%)	6 / 806 (0.7%)	3946.0	https://www.ebi.ac.uk/jdispatcher/psa/emboss_needle/summary?jobId=emboss_needle-I20250719-113601-0472-9120477-p1m
**HRAS**	189	188 / 189 (99.5%)	188 / 189 (99.5%)	0 / 189 (0%)	978.0	https://www.ebi.ac.uk/jdispatcher/psa/emboss_needle/summary?jobId=emboss_needle-I20250719-115058-0156-65006946-p1m
**TP53**	396	304 / 396 (76.8%)	326 / 396 (82.3%)	9 / 396 (2.3%)	1558.0	https://www.ebi.ac.uk/jdispatcher/psa/emboss_needle/summary?jobId=emboss_needle-I20250719-115335-0263-32878853-p1m

The high conservation of HRAS and FGFR3 between mouse and human proteins supports their use as reliable models for docking. Although TP53 showed slightly lower identity, the conserved regions remain sufficient for meaningful interaction analysis. Minimal gaps across alignments indicate structural integrity, reinforcing the appropriateness of mouse proteins in representing human targets. These results validate the use of these sequences for molecular docking.

## Molecular docking analysis

### FGFR3

**Fig. 4:**
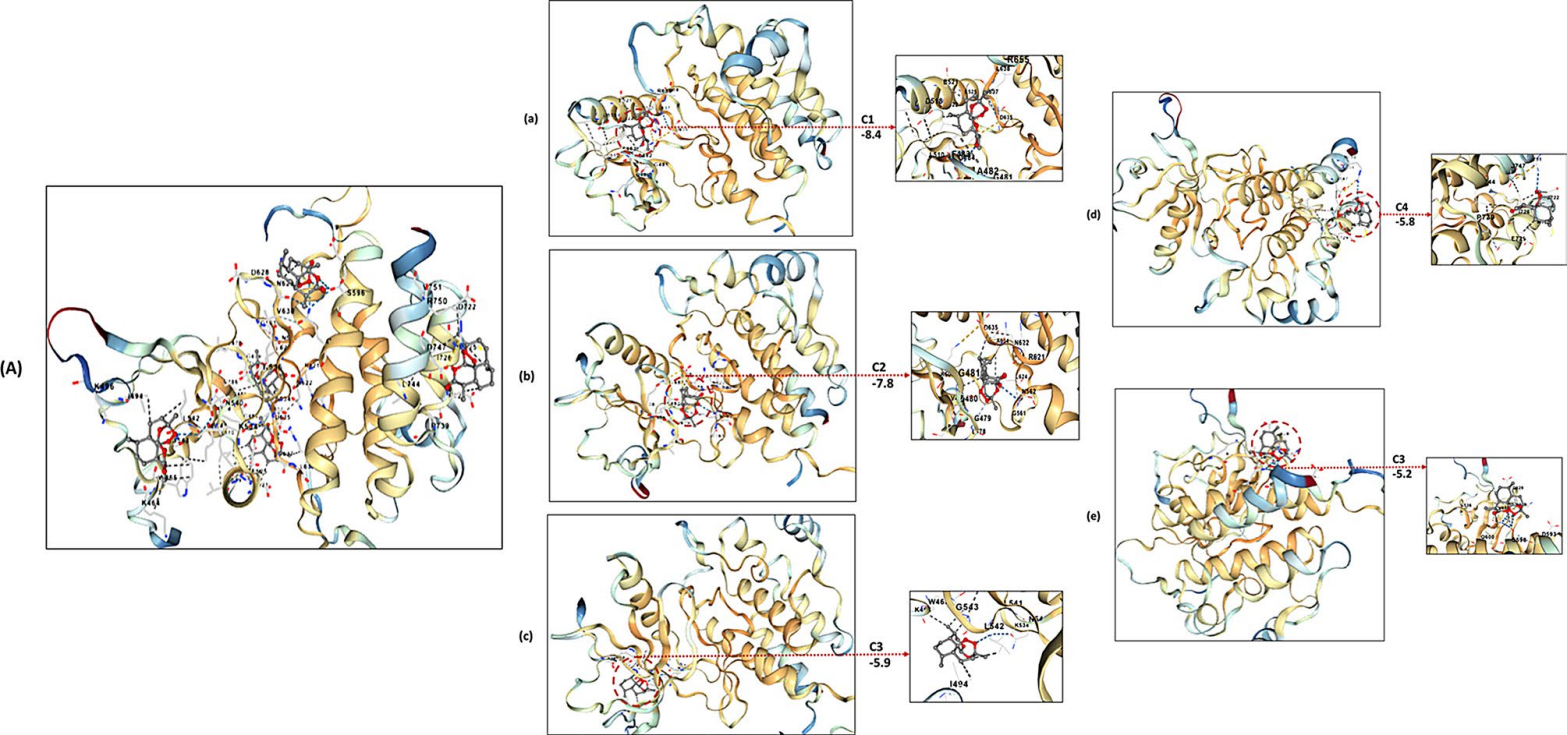
Molecular Docking visualization of artemisinin’s binding poses within the FGFR3 Active Site Using CB-Dock. This figure illustrates the results of the molecular docking analysis of the top five predicted binding poses of Artemisinin within the highest-affinity binding site (C1, Vina Score = -8.4 kcal/mol) of FGFR3. (**A**) A composite view showing the overlay of all five poses within the binding pocket. (**a**-**e**) Detailed 2D and 3D representations of each individual pose, highlighting specific hydrogen bonds (dashed lines) and other interactions with key amino acid residues

Molecular docking using the CB-Dock platform revealed that Artemisinin binds effectively to the FGFR3 kinase domain. Screening identified five potential pockets, with Site C1 being the most energetically favorable (-8.4 kcal/mol), located at the ATP-binding site. Detailed analysis of docking poses (Fig. 4; Table 5) showed that pose a is stabilized by hydrogen bonds with Gln535 and Asn537, while hydrophobic contacts with Ala482 and Phe531 further reinforce binding. Other poses highlight interactions with the hinge region (Gly481), the DFG motif (Asp593, Asn666), and ATP-coordinating residues (Lys494, Lys496). This computational analysis indicates a potential binding mechanism of Artemisinin with FGFR3 and suggests it may modulate its activity, though no direct experimental validation has been performed.

**Table 5:** Comparative molecular docking analysis of artemisinin with FGFR3, HRAS, and TP53 proteins using CB-Dock

Protein	Binding Site Code	Vina Score (kcal/mol)	Cavity Volume (Å³)	Box Size (x, y, z)	Center Coordinates (x, y, z)
FGFR3	C1	-8.4	1750	18, 26, 27	10, 31, 27
	C2	-7.8	703	18, 18, 18	3, 39, 18
	C3	-5.9	213	18, 18, 18	6, 50, 18
	C4	-5.8	587	18, 18, 18	-15, 40, 18
	C5	-5.2	259	18, 18, 18	-16, 44, 18
HRAS	C1	-7.3	906	29, 25, 30	-29, 19, 18
	C2	-6.6	74	18, 18, 18	-40, 22, 18
	C3	-5.7	84	18, 18, 18	-50, 13, 18
	C4	-5.7	67	18, 18, 18	-38, 19, 18
	C5	-4.7	142	18, 18, 18	-47, 6, 18
TP53	C1	-8.6	2902	30, 27, 18	62, 32, 76
	C2	-8.2	793	18, 18, 18	43, 23, 86
	C3	-7.2	881	25, 18, 18	69, 43, 84
	C4	-6.4	2676	18, 28, 18	32, 12, 85
	C5	-6.4	438	18, 18, 18	55, 25, 89

This table summarizes the top five predicted binding sites for Artemisinin on FGFR3, HRAS, and TP53 proteins, as determined by molecular docking simulations using CB-Dock. Key parameters include the Vina score (kcal/mol), where more negative values indicate stronger binding affinity, along with cavity volume (Å³), box size (Å), and center coordinates (Å) for each identified binding pocket

### HRAS

**Fig. 5:**
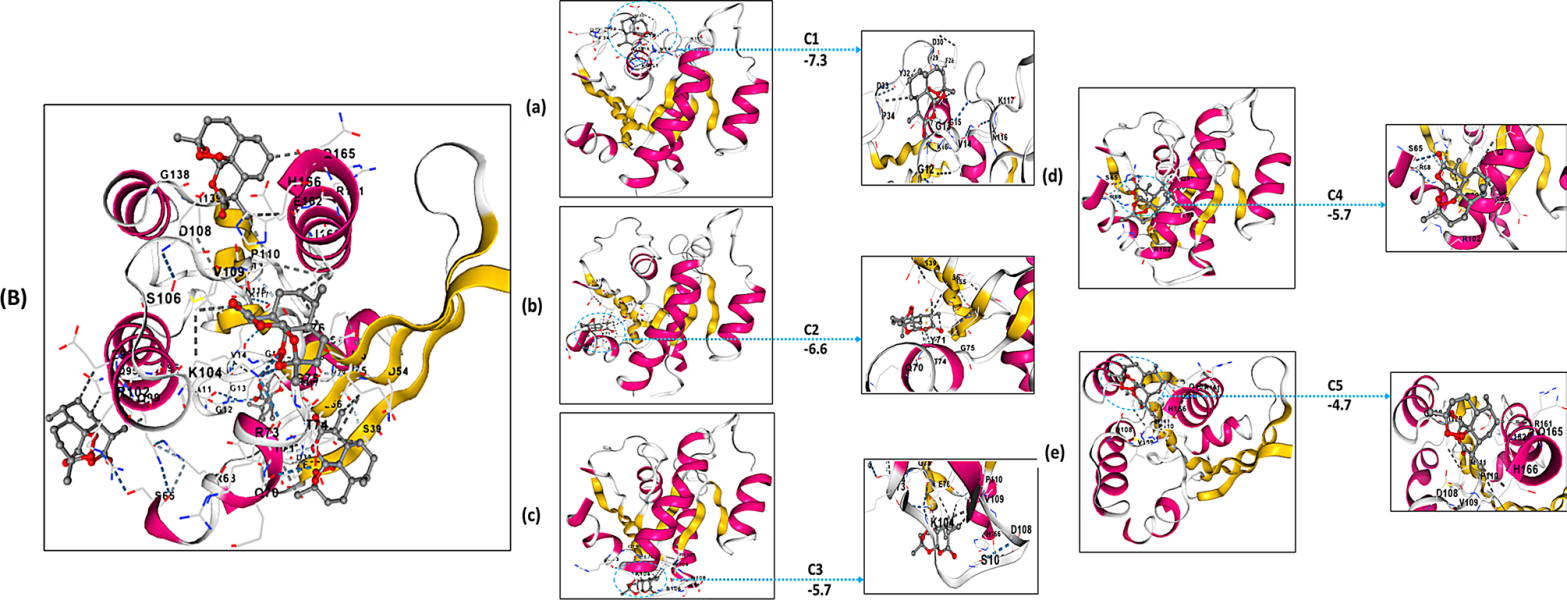
Molecular docking visualization of artemisinin’s binding poses within the HRAS active site using CB-Dock. This figure illustrates the results of the molecular docking analysis of the top five predicted binding poses of Artemisinin within the highest-affinity binding site (C1, Vina Score = -7.3 kcal/mol) of HRAS. (**A**) A composite view showing the overlay of all five poses within the binding pocket. (**a**-**e**) Detailed 2D and 3D representations of each individual pose, highlighting specific hydrogen bonds (dashed lines) and other interactions with key amino acid residues.

Docking simulations indicated that Artemisinin exhibits strong and stable binding to HRAS. Out of five predicted sites, Site C1 showed the highest binding affinity (-7.3 kcal/mol) and a cavity volume of 906 Å³. Figure 5 illustrates the top five poses (Fig. 5; Table 5), revealing Pose a with hydrogen bonds to G138 and D108, and hydrophobic interactions with S106 and P100. Pose b engages S65 (part of the P-loop) and Y96, while Pose d interacts with residues in the switch I region (Y32, P34). These diverse interactions imply Artemisinin may influence HRAS’s conformational flexibility and GTP-binding properties. Binding to the phosphate loop and switch I region could potentially affect HRAS function by modulating GTP binding and the conformational changes required for downstream signaling. These docking results imply that Artemisinin could interact with HRAS and potentially influence its conformational flexibility, based solely on computational predictions rather than experimental evidence.

### TP53

**Fig. 6:**
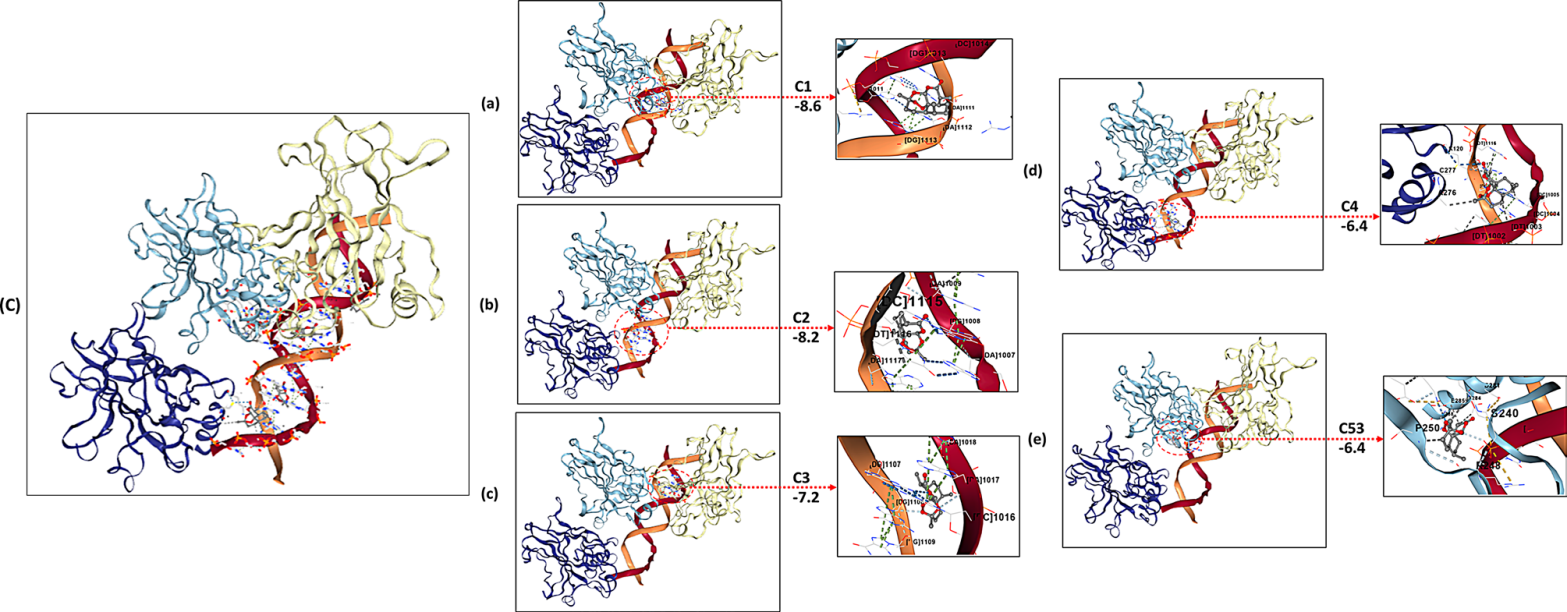
Molecular docking visualization of artemisinin’s binding poses within the TP53 protein using CB-Dock. This figure illustrates the results of the molecular docking analysis of the top five predicted binding poses of Artemisinin within the highest-affinity binding site (C1, Vina Score = -8.6 kcal/mol) of TP53. (**A**) A composite view showing the overlay of all five poses within the binding pocket. (**a**-**e**) Detailed 2D and 3D representations of each individual pose, highlighting specific hydrogen bonds (dashed lines) and other interactions with key amino acid residues

Artemisinin demonstrated high-affinity binding to TP53 as determined by CB-Dock analysis. Among five potential pockets, Site C1 presented the most favorable Vina score (-8.6 kcal/mol) and a large cavity (2902 Å³). Figure 6; Table 5 shows five major docking poses, with Pose a forming hydrogen bonds with C277 and S240, along with hydrophobic contacts involving R248 and L278. Other poses interact with cysteine and serine residues critical for structural integrity (C176, S179, C111, S182), and with residues in the DNA-binding domain. These interactions suggest that Artemisinin could stabilize TP53 and potentially affect its regulatory functions.

The in-silico findings suggest a possible stabilizing interaction of Artemisinin with TP53 and potential modulation of its regulatory functions, without direct confirmation from experimental studies.

## Quantitative RT-PCR analysis of gene expression in urinary bladder tissues

### FGFR3 and HRAS oncogenes

**Fig. 7:**
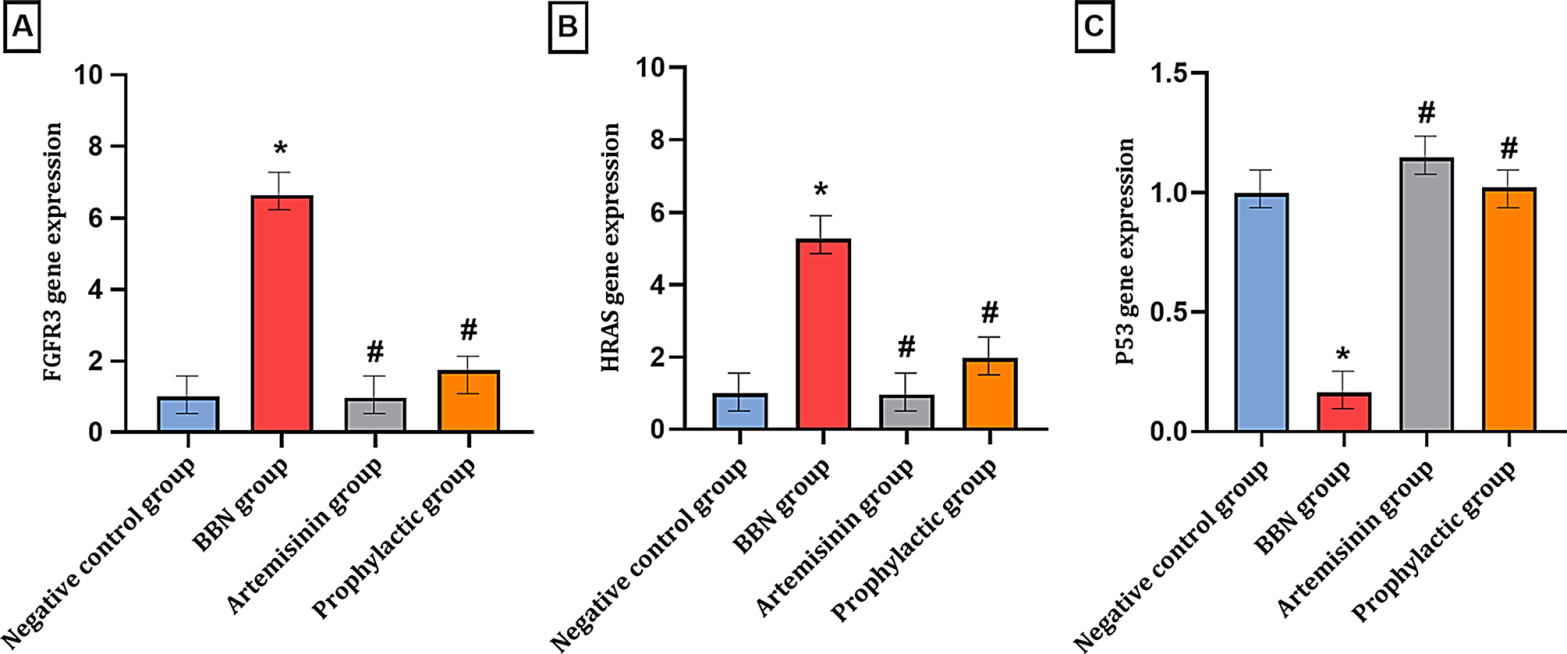
RT-qPCR was applied to detect the expressions of oncogenes (**A**) **FGFR3**, (**B**) **HRAS** and tumor suppressor genes (**C**) **P53**, in mouse urinary bladder tissues. Data are presented as mean ± SD from independent biological replicates (*n* = 10 per group) Statistical significance was determined by one-way ANOVA followed by LSD post-hoc test. Where, *P < 0.05 vs. Control, #*P* < 0.05 vs. BBN

FGFR3 expression was notably upregulated in the BBN group, exhibiting a 5.65-fold increase compared to the negative control group. In contrast, the prophylactic group displayed a modest increase of 0.73-fold, while the Artemisinin group showed a slight downregulation of (~ 96% reduction, 0.04-fold) relative to the negative control group (Table 6; Fig. 7A). Notably, when compared to the BBN group, FGFR3 expression was significantly downregulated, with a 5.68-fold reduction in the Artemisinin group and a 4.91-fold reduction in the prophylactic group.

**Table 6:** Analysis of gene expression in the experimental animal groups

Test	Group	Negative control group (A)	BBN group (B)	Artemisinin group (C)	Prophylactic group (D)
Gene expressions					
FGFR3	M ± SD	1.000 ± 0.000	6.649 ± 2.777^*^	0.967 ± 0.060^#^	1.735 ± 0.541^#^
HRAS	M ± SD	1.000 ± 0.000	5.263 ± 2.515^*^	0.954 ± 0.003^#^	1.963 ± 0.020^#^
P53	M ± SD	1.000 ± 0.000	0.163 ± 0.024^*^	1.147 ± 0.150^#^	1.022 ± 0.056^#^

Values are expressed as the mean ± SD from independent biological replicates (*n* = 10 per group) of the experiment. Statistical significance was determined by one-way ANOVA followed by LSD post-hoc test, Significant differences were analyzed using one-way ANOVA, where *P < 0.05 vs. the negative control group and #*P* < 0.05 vs. the BBN group

Similarly, HRAS expression was significantly elevated in the BBN group, with a 4.26-fold increase relative to the negative control group. In contrast, the prophylactic group exhibited only a minor increase of 0.96-fold, whereas the Artemisinin group showed a marked downregulation of (95% reduction, 0.046-fold) compared to the negative control group (Table 6; Fig. 7B). When compared to the BBN group, HRAS expression was significantly reduced, with a 4.31-fold decrease in the Artemisinin group and a 3.3-fold reduction in the prophylactic group. Overall, FGFR3 and HRAS expression was markedly increased in the BBN group, whereas no significant elevation was observed in the Artemisinin-treated or prophylactic groups, indicating the potential inhibitory effect of Artemisinin on these oncogenes.

### TP53 tumor suppressor gene

In the BBN group, P53 expression was significantly reduced, showing a 0.84-fold decrease compared to the negative control group. However, both treatment groups exhibited an upregulation in P53 expression, with (15% increase, 0.15-fold) and (2% increase; 0.022-fold) increases in the Artemisinin and prophylactic groups, respectively, compared to the negative control group. Notably, when compared to the BBN group, P53 expression was substantially upregulated, with 0.98-fold and 0.86-fold increases in the Artemisinin and prophylactic groups, respectively (Table 6; Fig. 7C). In contrast, P53 expression was reduced in the BBN group but substantially upregulated in both Artemisinin-treated and prophylactic groups, suggesting a protective effect of Artemisinin treatment.

## Histological findings

### Urinary bladder tissues

Artemisinin-treated mice showed no evidence of toxicity or abnormal behavior during the experimental period. Body weight and survival rates were comparable to control groups. Histological examination of urinary bladder tissues in negative control group revealed an intact urothelial lining with no pathological alterations, an unremarkable musculosa layer, and an absence of inflammatory cellular infiltrates within the submucosa at 16 weeks (Fig. 9A).

**Fig. 8:**
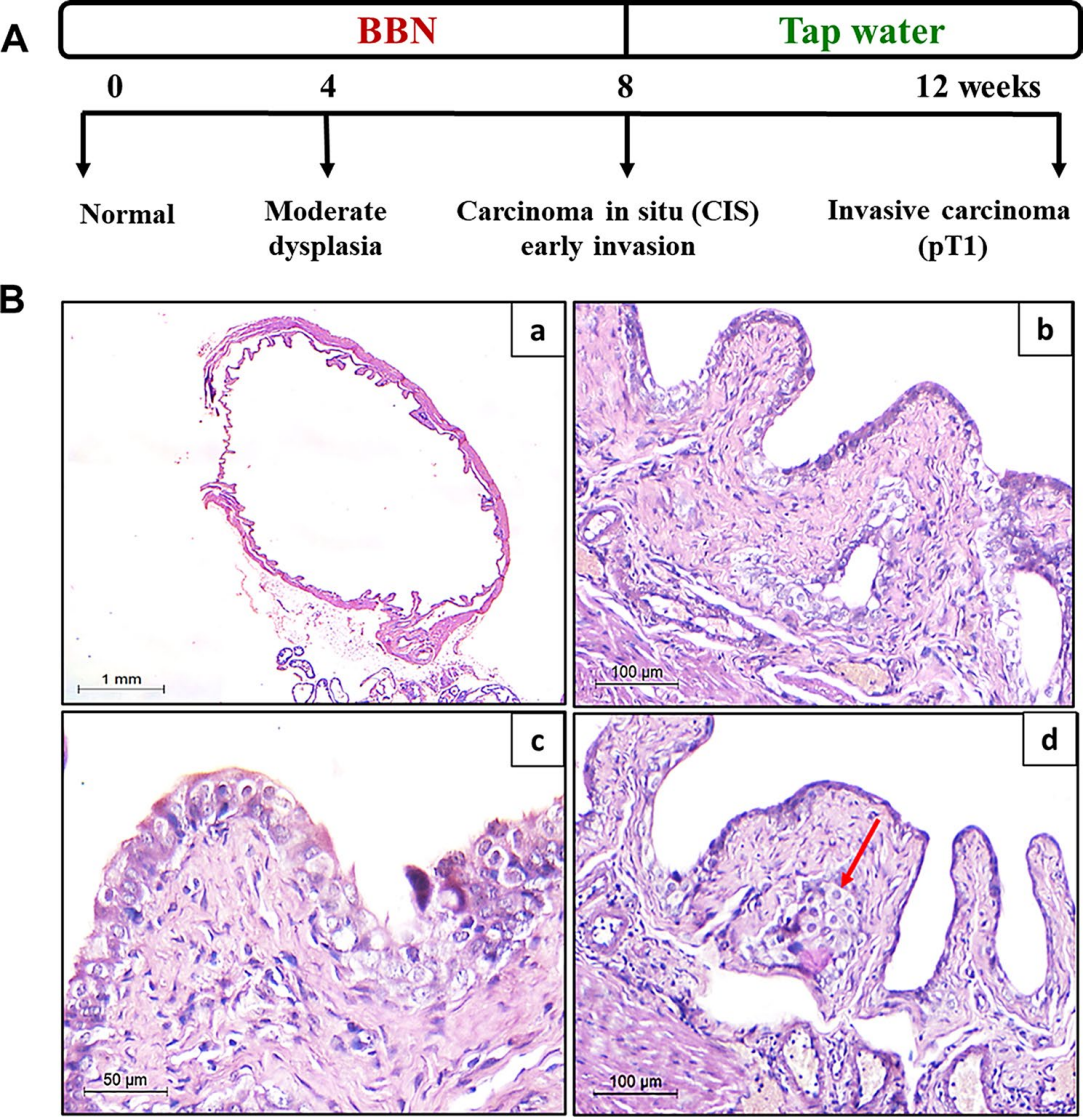
Histopathological examination of urinary bladder tissues across study groups. **(A) Negative control group**: Normal urothelial lining with no pathological changes or inflammation at 16 weeks (200×, scale bar = 200 μm). Inset: A high-power view showing regular maturation pattern. **(B) BBN group**: Carcinoma in situ with full-thickness dysplasia, nuclear pleomorphism, hyperchromasia, and invasive urothelial carcinoma (pT1) in the lamina propria at 12 weeks (100×, scale bar = 100 μm). Inset: A high-power view showing CIS. **(C) Artemisinin group**: Normal urothelial lining with mild inflammation in the lamina propria and submucosa at 16 weeks (200×, scale bar = 200 μm). Inset: a high-power view of the urothelium. and (**D) Prophylactic group**: Mild to moderate inflammation with preserved urothelial structure at 16 weeks (100×, scale bar = 100 μm). All sections were stained with H&E

**Fig. 9:**
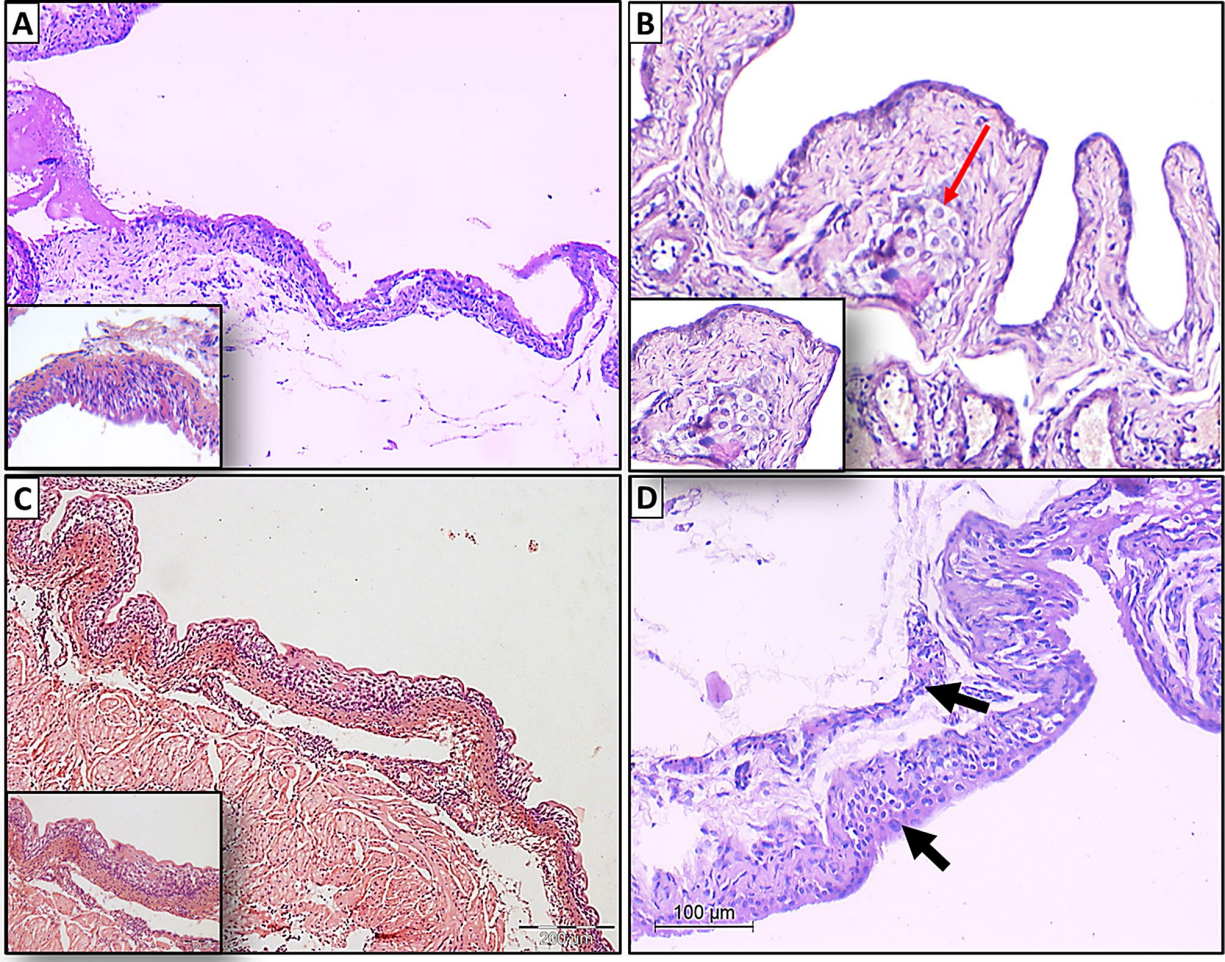
Histopathology of BBN-induced bladder cancer in mice. **A**, Schematic illustration of the BBN treatment timeline. **B**, Representative H&E-stained bladder sections showing progressive histological alterations: **(a)** Normal urothelium with no morphological changes (control) (1×, scale bar = 1 mm), **(b)** Moderate dysplasia with broad papillae lined by thickened urothelial epithelium at week 4 (100×, scale bar = 100 μm), **(c)** Full-thickness urothelial dysplasia consistent with carcinoma in situ (CIS) at week 8 (50×, scale bar = 50 μm) and **(d)** Invasive carcinoma nest (pathologic stage pT1) infiltrating the lamina propria at week 12 (100×, scale bar = 100 μm) (red arrow)

To investigate the sequential urothelial changes associated with BBN-induced urothelial carcinoma, urinary bladder tissue samples were collected from male mice at various time points: before BBN exposure (control group), during treatment with BBN, and after cessation of BBN administration. In BBN group (Fig. 8B), full-thickness urothelial dysplasia was observed after 4 weeks of exposure, progressing to features consistent with carcinoma in situ (CIS) by the 8th week. These cytomorphological changes were characterized by marked nuclear and cellular pleomorphism, an increased nucleo-cytoplasmic ratio, and prominent hyperchromasia. By the 12th week, invasive nests of neoplastic urothelial cells were detected within the lamina propria, indicating the progression to a high-grade invasive urothelial carcinoma (Pathologic stage pT1), according to [34]. Importantly, no evidence of invasion into the muscularis propria, or lymphovascular space was observed (Fig. 9B). Conversely, Artemisinin group exhibited an unremarkable urothelial lining with no dysplastic changes, accompanied only by mild inflammatory infiltrates within the lamina propria and submucosa at 16 weeks (Fig. 9C). Notably, in prophylactic group, which received artemisinin prior to BBN exposure, mild to moderate mononuclear inflammatory infiltrates were present in the lamina propria and submucosa at 16 weeks (Fig. 9D). The urothelium maintained preserved cell polarity, with only mild nuclear pleomorphism in the superficial layers, while the basal layers remained uniform and free from atypia.

### Kidney tissues

**Fig. 10:**
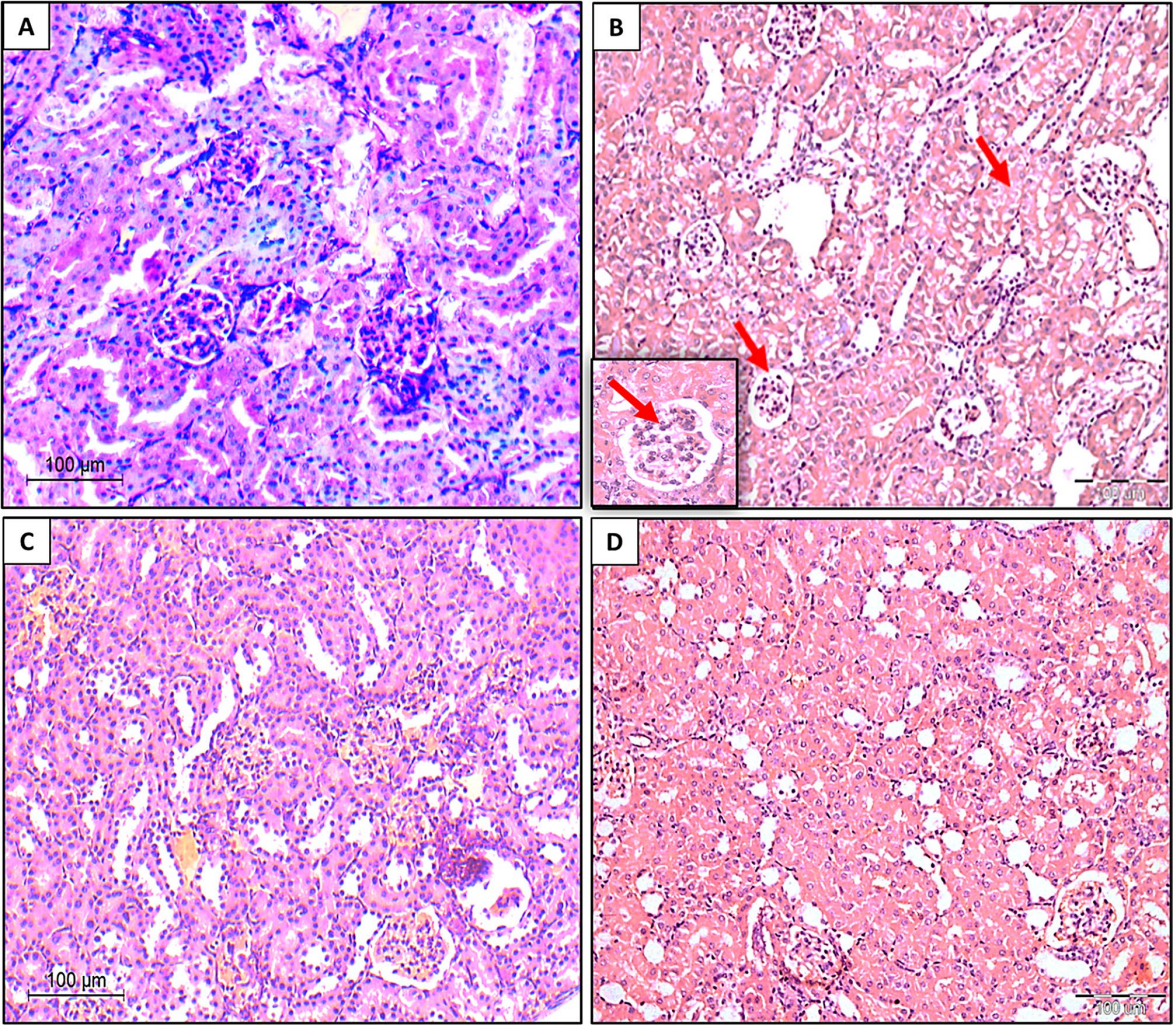
Histological examination of kidney tissues across study groups. **(A)** Negative control group: Normal kidney architecture with no pathological changes at 16 weeks. **(B) BBN group**: Significant alterations including interstitial nephritis, tubular swelling, casts, and mesangial hypercellularity at 12 weeks. Inset: High-power view showing prominent mesangial hypercellularity and tubular epithelial cell degeneration. **(C) Artemisinin group**: Preserved kidney structure with no signs of inflammation or damage at 16 weeks. and **(D) Prophylactic group**: Normal kidney structure with mild interstitial inflammation at 16 weeks. All sections were stained with H&E and observed at 100× magnification (scale bar = 100 μm)

Histological examination of kidney tissues across the study groups revealed distinct findings. Where, in the negative control group, kidney sections exhibited normal histological features, including patent glomeruli and well-preserved renal tubules. No interstitial inflammatory infiltrates, fibrosis, or vascular abnormalities were observed, confirming the absence of pathological changes at 16 weeks (Fig. 10A). In contrast, BBN group exhibited interstitial nephritis, characterized by prominent lymphoplasmacytic infiltration within the interstitial spaces. Tubular changes, such as cloudy swelling and the presence of tubular casts, were also observed. Furthermore, the glomeruli showed mesangial hypercellularity at 12 weeks (Fig. 10B). Artemisinin group displayed normal kidney morphology, with patent glomeruli and intact renal tubules. There were no vascular changes, interstitial inflammation, or tubular casts present, indicating the preservation of renal structure at 16 weeks (Fig. 10C). In comparison, in prophylactic group, kidney architecture appeared largely normal, with patent glomeruli and unremarkable renal tubules. Mild interstitial inflammation was observed, although no significant vascular changes or tubular casts were present at 16 weeks (Fig. 10D).

## Biochemical and hematological assays

### Biochemical analysis of Urea and Creatinine

The biochemical analysis of urea and creatinine revealed a significant increase in the BBN group, with both parameters elevated compared to the negative control group (*P* < 0.05). In contrast, the Artemisinin and prophylactic groups showed no significant changes in urea and creatinine levels relative to the A group (*P* > 0.05).

**Fig. 11:**
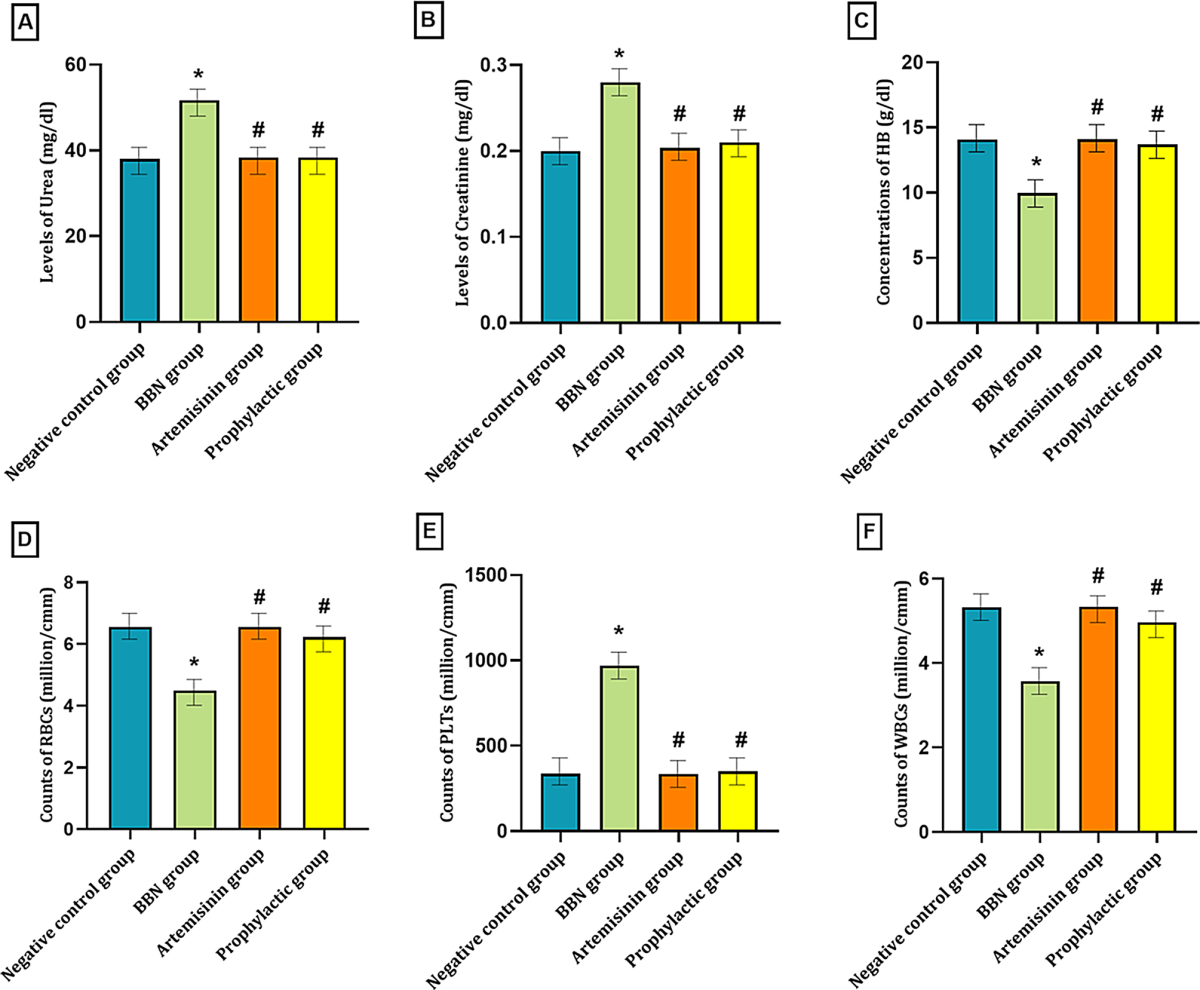
Biochemical and hematological analysis in studied groups. (**A**) urea levels, (**B**) **creatinine levels**, (**C**) **Hb concentrations**, (**D**) **RBCs count**, (**E**) **PLTs count** and (**F**) **WBCs count**. Data are expressed as mean ± SD from independent biological replicates (*n* = 10 per group), Statistical significance was determined by one-way ANOVA followed by LSD post-hoc test, where *P < 0.05 vs. Control, #*P* < 0.05 vs. BBN

However, when compared to the BBN group, both the Artemisinin and prophylactic groups exhibited a significant reduction in urea and creatinine levels (*P* < 0.05), as presented in Table 7 and illustrated in Fig. 11.

**Table 7:** Biochemical and hematological analysis of the experimental animal groups

Test	Group	Negative control group (A)	BBN group (B)	Artemisinin group (C)	Prophylactic group (D)
**Biochemical analysis**					
Urea	M ± SD	38.00 ± 1.000	51.666 ± 5.0332^*^	38.333 ± 1.154^#^	38.333 ± 1.527^#^
Creatinine	M ± SD	0.200 ± 0.010	0.280 ± 0.020^*^	0.203 ± 0.020^#^	0.210 ± 0.010^#^
**Hematological analysis**					
HB	M ± SD	14.083 ± 0.485	9.966 ± 0.407^*^	14.100 ± 0.150^#^	13.700 ± 0.010^#^
RBCs	M ± SD	6.550 ± 0.117	4.496 ± 0.310^*^	6.553 ± 0.136^#^	6.223 ± 0.639^#^
PLTs	M ± SD	338.333 ± 10.408	969.00 ± 30.446^*^	336.00 ± 9.848^#^	351.0 ± 4.000^#^
WBCs	M ± SD	5.323 ± 0.158	3.570 ± 0.615^*^	5.336 ± 0.072^#^	4.963 ± 0.015^#^

Values are expressed as the mean ± SD from independent biological replicates (*n* = 10 per group) of the experiment. Statistical significance was determined by one-way ANOVA followed by LSD post-hoc test, Significant differences were analyzed using one-way ANOVA, where *P < 0.05 vs. the negative control group and #*P* < 0.05 vs. the BBN group

### Hematological parameters analysis

The hematological analysis revealed significant alterations in the BBN group, with hemoglobin (HB), red blood cells (RBCs), and white blood cells (WBCs) significantly reduced compared to the negative control group. In contrast, platelet (PLT) levels showed a substantial increase, rising approximately 2.86-fold relative to the negative control group (*P* < 0.05). In the Artemisinin and prophylactic groups, hematological parameters were largely similar to those of the negative control group (*P* > 0.05), with no significant deviations observed. However, when compared to the BBN group, both Artemisinin and prophylactic groups exhibited significant improvements in HB, RBC, and WBC counts (*P* < 0.05). Conversely, PLT counts were significantly reduced in both the Artemisinin and prophylactic groups (*P* < 0.05).as shown in Table 7 and illustrated in Fig. 11.

## Discussion

In this study, we investigated the prophylactic potential of artemisinin in preventing bladder carcinogenesis induced by N-butyl-N-(4-hydroxybutyl) nitrosamine (BBN) in a mouse model. By integrating bioinformatics (STRING protein interaction network), molecular docking, gene expression analysis, histopathology, and systemic biomarkers, we demonstrate that artemisinin prevents early oncogenic events and mitigates tissue and systemic damage typically observed in bladder cancer development. Notably, Prophylactic administration of Artemisinin prior to carcinogen exposure showed potential protective effects, highlighting its role as a preventive agent. Protein–protein interaction analysis using the STRING database identified FGFR3, HRAS, and TP53 as central hubs in bladder cancer signaling, particularly within the MAPK and PI3K/AKT pathways [35, 36]. These proteins regulate essential cellular processes such as proliferation, differentiation, and apoptosis, and their dysregulation is a hallmark of urothelial carcinogenesis. Specifically, the aberrant activation of FGFR3 and HRAS, coupled with suppression or mutation of TP53, represents a critical molecular triad driving early tumor initiation and progression [35, 37, 38]. This highlights their relevance as strategic targets for early prophylactic interventions. Molecular docking analysis supported this premise by demonstrating that artemisinin exhibits strong and specific binding affinities to catalytically active and functionally relevant domains of FGFR3 and HRAS. These interactions suggest that artemisinin may disrupt downstream oncogenic signaling cascades. In addition, artemisinin showed a favorable interaction profile with TP53, potentially contributing to the reactivation of its tumor suppressor functions. Collectively, these in silico findings provide a mechanistic foundation for artemisinin’s direct interference at the protein level, which may precede transcriptional modulation and contribute to a preemptive antitumor effect. However, these docking results should be interpreted with caution, as they represent predictive in silico interactions and require further experimental validation to confirm binding specificity and biological relevance. These computational predictions were validated through in vivo qRT-PCR expression analysis, which revealed significant gene-level modulation in the prophylactic group. In mice exposed to BBN, FGFR3 and HRAS expression levels were markedly elevated (5.65- and 4.26-fold increases, respectively), while TP53 expression was downregulated by 0.84-fold, consistent with enhanced proliferative signaling and impaired genomic stability [27, 35, 38]. In contrast, prophylactic administration of artemisinin reversed these molecular changes: FGFR3 and HRAS were downregulated by 5.68- and 4.31-fold, respectively, and TP53 was upregulated by 0.98-fold. These changes suggest that artemisinin not only counteracts the oncogenic transcriptional shift induced by BBN but also partially restores the tumor-suppressive environment in the urothelium. These results are in line with previous studies demonstrating that dihydroartemisinin suppresses FGFR3 and HRAS expression and induces apoptosis in cancer models, and that Artemisia annua extracts enhance TP53 expression in breast and gastric cancers through ERK1/2-mediated pathways [26, 39, 40]. Altogether, the integration of protein interaction, molecular docking, and gene expression data provides a compelling mechanistic explanation for the prophylactic efficacy of artemisinin, suggesting its potential role in modulating early molecular events in bladder cancer development. Bearing in mind that the molecular alterations in cancer oncogenesis occur before morphological changes are even apparent microscopically, since the main aim of this work was to highlight the preventive effect of Artemisinin in urothelial carcinoma. The morphological pictures captured at the studied time intervals were performed at later stages where in situ epithelial changes and even invasive carcinoma had already occurred. Therefore, the histological changes demonstrated in the study are to confirm that carcinogenic events have occurred. The molecular changes translated into notable histological protection in the bladder. As expected, prolonged BBN exposure led to urothelial dysplasia by week 8 and invasive carcinoma by week 12 [26, 41]. In contrast, prophylactic artemisinin preserved bladder architecture, with only mild inflammatory infiltrates and an absence of dysplasia or neoplasia. These findings demonstrate that artemisinin acts before histological transformation occurs, supporting the concept that chemoprevention is most effective during early, molecular phases of tumor development. Comparable studies using ω-3 supplementation have shown similar modulation of early-stage dysplasia without full tumor suppression [42], while artesunate significantly reduced tumor volumes in advanced bladder cancer models [43]. Kidney histology further confirmed artemisinin’s systemic protective effects. While BBN-treated mice showed signs of tubulointerstitial nephritis and mesangial hypercellularity [26], the prophylactic group maintained intact renal architecture. Biochemical markers mirrored these findings, with BBN-induced elevations in serum urea and creatinine significantly attenuated in the prophylactic group (*P* < 0.05), indicating preserved renal function. These results are supported by earlier studies reporting renoprotective effects of artemisinin derivatives in models of nephrotoxicity, including those induced by cisplatin, diabetes, and oxidative stress [44–49]. Hematological profiles also improved with prophylactic artemisinin. BBN exposure led to reductions in hemoglobin, red and white blood cell counts, and a concurrent increase in platelets, reflecting systemic inflammation and hematopoietic suppression. Artemisinin restored these parameters toward normal levels, suggesting a stabilizing effect on immune and hematopoietic function [26, 50]. The selected dose of artemisinin (200 mg/kg) was based on prior studies demonstrating both efficacy and safety. Consistent with clinical data indicating tolerability at equivalent human doses, no signs of systemic or neurotoxicity were observed in our model [29, 51]. These findings support the translational potential of artemisinin as a prophylactic agent against early-stage urothelial carcinoma. While promising, these results are derived from a murine model; therefore, translation to humans should be approached cautiously. Clinical trials in high-risk populations will be essential to confirm safety, efficacy, and applicability in real-world settings. These findings further reinforce artemisinin’s role in mitigating systemic complications associated with early carcinogenesis. Together, these findings present a coherent and compelling case for the prophylactic role of artemisinin in bladder cancer prevention. Administered prior to carcinogenic insult, artemisinin effectively suppresses key oncogenes, restores tumor suppressor gene expression, preserves tissue architecture, and prevents biochemical and hematological abnormalities associated with BBN-induced urothelial carcinogenesis. The convergence of in silico modeling, gene expression analysis, histopathological evaluation, and systemic biomarker assessment strongly supports its efficacy in early intervention. Notably, the superior outcomes observed in the prophylactic group underscore the critical importance of timely administration, as intervention after molecular dysregulation may diminish therapeutic benefit. These results establish artemisinin as a promising, low-toxicity prophylactic agent for early-stage bladder cancer. Despite these encouraging findings, the study has certain limitations. Protein expression and functional validation were not assessed, which may provide additional mechanistic insights. Moreover, the use of a mouse model inherently limits direct extrapolation of results to humans, as interspecies differences may affect drug metabolism and tumor biology. While the present study focused on RT-qPCR analysis, future work will aim to include protein-level validation using Western Blot and Immunohistochemistry to further confirm the drug’s effects, providing a more comprehensive understanding of its molecular impact. Further clinical investigations are warranted to validate these findings and explore its potential application in high-risk human populations.

## Conclusion

This study demonstrates that artemisinin, when used prophylactically, can effectively prevent BBN-induced bladder cancer in mice. Through integrated analyses-STRING interaction mapping, molecular docking, and qRT-PCR-artemisinin was shown to modulate key genes (FGFR3, HRAS, TP53) involved in early urothelial carcinogenesis. These molecular effects were supported by preserved bladder and kidney histology and normalized biochemical and hematological profiles, indicating systemic protection. Together, these findings highlight artemisinin’s potential as a prophylactic agent against early-stage bladder cancer, supporting its further evaluation in high-risk human populations. Future work will extend these findings by assessing protein expression using Western Blot and Immunohistochemistry, providing a more comprehensive understanding of the drug’s impact.

## Data Availability

The datasets generated and/or analysed during the current study are publicly available from the following repositories and resources:- FGFR3:UniProt: [https://www.uniprot.org/uniprotkb/Q61851/entry NCBI Gene: 14184 [https://www.ncbi.nlm.nih.gov/gene/14184 PDB: [https://www.rcsb.org/structure/4K3- HRAS: UniProt: [https://www.uniprot.org/uniprotkb/Q61411/entry NCBI Gene: 15461 [https://www.ncbi.nlm.nih.gov/gene/15461 PDB: [https://www.rcsb.org/structure/5P21- TP53:UniProt: [https://www.uniprot.org/uniprotkb/P02340/entry NCBI Gene: 22059 [https://www.ncbi.nlm.nih.gov/gene/22059 PDB: [https://www.rcsb.org/structure/1TSR- Artemisinin: PubChem CID: 68827 [https://pubchem.ncbi.nlm.nih.gov/- Additionally, sequence alignments and comparative analyses were performed using the EMBOSS Needle tool, available at: [https://www.ebi.ac.uk/Tools/psa/emboss_needle/All other relevant data supporting the findings of this study are included within the article.

## Abbreviations

BBN: N-butyl-N-(4-hydroxybutyl) nitrosamine
BCPN: N-butyl-N-(3-carboxypropyl) nitrosamine
CBC: Complete Blood Count
CIS: Carcinoma in Situ
Ct: Cycle Threshold
ErbB: Erythroblastic Leukemia Viral Oncogene Homolog
FRS: Fibroblast Growth Factor Receptor Substrate
H19: Histidine-rich protein 19
H&E: Hematoxylin and Eosin
HB: Hemoglobin
HRAS: Harvey-Ras
KRAS: Kristen-Ras
MAPK: Mitogen-Activated Protein Kinase
MIBC: Muscle-Invasive Bladder Cancer
N-Ras: Neuroblastoma-Ras
PT1: Pathologic stage
PLT: Platelets
PPI: Protein–protein interaction
PPP1R13B: Protein Phosphatase 1 Regulatory Subunit 13B
qRT-PCR: Quantitative Real-time PCR
RBCs: Red Blood Cells
RGL1: Ral Guanine Nucleotide Dissociation Stimulator-Like 1
RPS6KA2: Ribosomal Protein S6 Kinase A2
SHC2: SHC Adaptor Protein 2
SPSS: Statistical Package for Social Sciences
VEGF: Vascular Endothelial Growth Factor
WBCs: White Blood Cells
